# CircMCTP2 (has-circ-0000658) facilitates the proliferation and metastasis of bladder carcinoma through modulating the miR-498/murine double minute-2 axis

**DOI:** 10.1080/21655979.2022.2054161

**Published:** 2022-04-27

**Authors:** Qiao Gu, Wenjie Hou, Lijuan Shi, Zonghao Zhu, Huan Liu, Xiaozhou He

**Affiliations:** a Department of Gynecology and Obstetrics, The Third Affiliated Hospital of Soochow University, Changzhou, P.R. China; b Department of Gynecology and Obstetrics, Dushu Lake Hospital Affiliated to Soochow University (Medical Center of Soochow University), Suzhou, P.R. China; c Department of Pathology, Changzhou Hospital of Traditional Chinese Medicine, Changzhou, P.R. China; d Department of Urology, The Third Affiliated Hospital of Soochow University, Changzhou, P.R. China

**Keywords:** Bladder carcinoma, circMCTP2, miR-498, MDM2

## Abstract

CircMCTP2 is a novel circRNA, which is associated with various kinds of malignant tumors progression, such as gastric cancer. However, the function of circMCTP2 in bladder carcinoma (BC) has no idea. The purpose of this study was tantamount to functionally dissect circMCTP2 in the progression of BC. In our study, circMCTP2 expression was strongly increased in BC tissues and cell lines. High expression of circMCTP2 predicted a poor prognosis of BC patients. CircMCTP2 deficiency impaired the cell growth, migration as well as invasive ability of BC cell lines (J82 and T24). *In vivo*, circMCTP2 deficiency cut the tumor growth rates and the tumor weight. In BC cells, circMCTP2 deficiency enhanced the translation of E-cadherin, while diminishing the translation of N-cadherin, Vimentin, and Snail. Moreover, circMCTP2 acted as a sponge of miR-498 to regulate murine double minute-2 (MDM2) expression. In BC tissues, a negative correlation was observed between the expression levels of circMCTP2 and miR-498. Additionally, either miR-498 silencing or MDM2 over-expression augmented the carcinogenic action of circMCTP2 on BC. In conclusion, our study showed that circMCTP2 regulates the expression of MDM2 by sponging miR-498 to promote the development of BC. These findings offer a new strategy for early diagnosis of BC and its therapeutics.

## Graphical abstract

**Figure:**
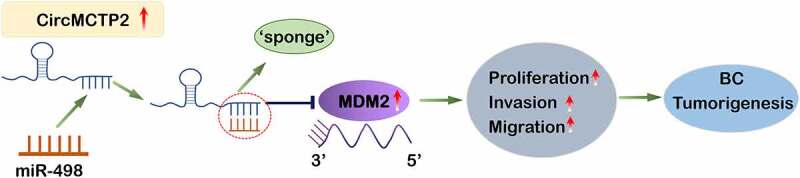

## Highlights

- CircMCTP2 is a novel circRNA and the function of circMCTP2 in BC is unknown.
- The high expression of circMCTP2 predicted a poor prognosis of BC patients
- CircMCTP2 regulates the expression of MDM2 by sponging miR-498 to promote the development of BC.
- These findings provide a new strategy for early diagnosis of BC and its therapeutics.

## Introduction

Bladder carcinoma (BC) is the most common malignant tumor of the urinary system, ranking the tenth most frequent tumor worldwide [1,2]. As per the American Cancer Society statistics, approximately 81, 400 new BC cases and 17, 980 deaths were recorded in the United States in 2020 [3]. Although many advances in therapeutic strategies have been made in the treatment of BC, such as surgical resection, chemotherapy, and radiation therapy [4], the 5-year survival rate of BC patients is still unsatisfactory because its high metastasis and recurrence rates [5]. However, our understanding of the molecular mechanisms of BC development is flimsy, which greatly stagnates the current BC treatment. Hence, it is imperative to molecularly dissect the BC progression and tumorigenesis, thereby identifying novel pharmaceutical targets for early diagnosis or therapeutics.

Circular RNAs (circRNAs) are emerged as a class of non-coding RNAs that is generated by back-splicing of a pre-mRNA [6]. CircRNA is widely expressed in tissues [7], and regulates various cellular processes, including cell proliferation, differentiation, senescence, metastasis, epithelial-mesenchymal transition, and apoptosis [8–11]. Under pathological conditions, the dysregulated cellular circRNAs contribute to the development of diverse disorders, including diabetes, pulmonary hypertension, cardiovascular diseases, and malignant tumors [12–15]. Increasing evidence demonstrates that the dysregulated circRNAs play an important role in the development of BC. For instance, circ_100984 regulates the miR-432-3p/c-Jun/YBX-1/β- catenin signaling pathway to promote the progression of BC [16]. CircCEP128 deficiency represses the progression of BC through modulating themicroRNA-515-5p/SDC1 Axis [17]. CircMYLK increases the expression of CCND3 to facilitate the proliferation and metastasis of BC cells through sponging miR-34a [18]. CircNT5E speeds up the progression of BC by sponging miR-502-5p [19]. CircZNF139 accelerates the cell proliferation and metastasis by activating the PI3K/AKT signaling in the BC [20]. CircMCTP2 is a novel circRNA located in chromosome 15, and is essential for the progression of gastric cancer [21]. However, the function of circMCTP2 in BC has not meant explored.

MicroRNAs (miRNAs) is a highly conserved member of the small noncoding RNAs and participates in diverse biological activities through suppressing the translation of mRNA [22]. Many studies have reported that miRNAs are dysregulated in many diseases, which is closely associated with the process of metastasis and proliferation in BC [23]. Recent studies implicated miR-498 in diverse tumor progression, such as hepatocellular cancer, lung cancer and colon cancer [24–26]. However, the role of miR-498 in the development of BC remains unknown. MDM2 is a p53-binding protein that negatively regulates p53 activity through the induction of protein degradation [27,28]. Moreover, it has been noted that miRNAs could directly target the MDM*2* gene to regulate tumor progression [29–31]. As such, MDM2 has been suggested as a promising target for cancer treatment [32]. For example, miR-15a suppresses the GATA2/MDM2 axis to inhibit the proliferation and invasiveness of osteosarcoma cells in vitro through the p53 signaling pathway [33,34]. In addition, the aberrant expression of MDM2 was linked to the development of BC [34–36]. However, the relationship between circMCTP2 and miR-498 or MDM2 has not meant fully elucidated.

Here in this study, we aim to investigate the function of circMCTP2 in BC progression. According to its high expression in BC, we hypothesized that circMCTP2 regulates BC progression as a competing endogenous RNA (ceRNA). Subsequently, we explored the intrinsic molecular mechanism of circMCTP2, in order to provide new strategies for the treatment of BC.

## Method and material

### Clinical tissue samples

Tumor tissues and adjacent noncancerous specimens were collected from 40 BC patients by surgery at The Third Affiliated Hospital of Soochow University. BC patients had not received any chemotherapy and radiotherapy before surgery. Cancer and noncancerous regions were identified by two professional pathologists. Tissue specimens were directly stored in liquid nitrogen (Delun, Shanghai, China) for RNA collection. The written informed consent was issued by all participants. This research was authorized by the Institutional Ethics Committee of The Third Affiliated Hospital of Soochow University (approval number: [KY-H-2020-12-18]) and was carried out in accordance with the Declaration of Helsinki [36].

### Cell culture and transfection

HEK-293 T cells were obtained from the Sciencell Company (Sciencell, USA). Five BC cell lines (SW780, J82, 5637, T24, and UMUC3) and normal urothelial cells line (SV-HU-1) were purchased from the ATCC Company (ATCC, USA). Cells were grown in RPMI‑1640 medium (Gibco, USA) containing 20% fetal bovine serum (FBS, Gibco, USA) at room temperature in a humidified atmosphere with 5% CO_2_. The sh-NC, sh-circMCTP2, mimics control, miR-498 mimics, inhibitor control as well as miR-498 inhibitor were purchased from Genechem Co.,Ltd (Shanghai, China). Cells were incubated in 6‑well plates and transfected with the indicated constructs with Lipofectamine 3000 Reagent (Invitrogen, USA) [37].

### Bioinformatic analysis

BC patient gene profiling data were downloaded from the National Center for Biotechnology Information Gene Expression Omnibus (GEO) database (GES92675 and GES140584) (https://www.ncbi.nlm.nih.gov/). Aberrant expression of circRNAs and miRNAs between the BC group and the para‐carcinoma group was identified (Fold‐change ≥ 2.0, P < 0.05) using a fold‐change cut off value. The sponged miRNAs by the circMCTP2 were predicted using the website tool CircInteractome (https://circ-interactome.nia.nih.gov/). The target mRNA of miR-498 was predicted by the online StarBase database (http://starbase.sysu.edu.cn) [38].

### RNA isolation and RNase R treatment

Cytoplasmic and Nuclear RNA Purification Kit (Norgen Biotek Corp, Canada) was used to extract cytoplasmic and nuclear RNAs. Total RNA from BC tissues and cell lines were extracted by using the TRIzol reagent (Thermo Fisher Scientific, USA) [39]. The total RNA of BC cell lines was treated with RNase R (3 U/mg) for 15 min at room temperature. QRT‐PCR analysis was carried out to quantify the mRNA expression of MCTP2 and circMCTP2 [40].

### Quantitative real-time PCR assay

Total RNA was reverse transcribed to cDNA by a Reverse Transcription kit (Thermo Fisher Scientific, USA). qPCR was performed on a Thermal Cycler Dice Real Time system (Takara, Japan) with SYBR Green PCR master mix (Takara, Japan). Experiment was repeated three times and the relative gene expression was measured with 2^−ΔΔCt^ method [41]. The primers utilized for qRT-PCR were presented in Table 2.

**Table 2.** Primers used for qPCR

Gene	Forward 5′–3′	Reverse 5′–3′
CircMCTP2	5′-ACCAGAAGAGCCAGAGGAGTC-3′	5′-TGGCCTGGTCCGCTGTTTTAA-3′
MiR-498	5′-UAAUGGTCGCGUCCGGGTCC-3′	5′-GUUGUGGACCATATAGUAAAUGU-3′
MDM2	5′-TACTCAACGAGACCGCCCAA-3′	5′-CAGTCCACTGAGAACAGGAC-3′
U6	5′-CTCGCTTCGGCAGCACA-3′	5′-AACGCTTCACGAATTTGCGT-3′
GAPDH	5′-GCATCCTGGGCTACACTG-3′	5′-ACTTCAGGAGCATCTGAAATAGGT-3′

### Western blot analysis

Protein extraction from BC cells was carried out following a previous study [37]. Briefly, the protein from carcinoma cell lines was collected with RIPA lysis buffer (Sigma-Aldrich. USA) supplemented with protease inhibitor cocktail (PIC, Roche, USA). Proteins were separated on a 12% SDS-PAGE gel and transferred to a PVDF membrane (Millipore, USA). Then, the membrane was blocked with 5% BSA at 37°C for 2 h. After wash with PBS, the membrane was incubated with primary antibodies against Snail (1:1000, CST, USA), N-cadherin (1:1000, Abcam, USA), E-cadherin (1:2000, Abcam, USA), Vimentin (1:2000, CST, USA), MDM2 (1:2000, CST, USA) as well as GAPDH (1:2000, Proteintech, USA) at 4°C for 24 h. Next, the membrane was incubated with secondary antibody (1:10,000, Jackson, USA) at 37°C for 2 h. Blots were detected with ECL reagents (Amersham, UK) and assessed with ImageJ software (the National Institutes of Health, USA).

### CCK‑8 assay

Cell proliferation capability was measured with the CCK‑8 assays. Briefly, cells were transfected with the indicated constructs for 72 h. Then the cells were harvested and seeded into 96‑well plates at a density of 1 × 10^4^ cells/well. Followed by incubation for 0 h, 24 h,48 h, and 72 h. Subsequently, 10 uL of CCK‑8 reagents (Beyotime, Shanghai, China) was added to each well and incubated with cells at 37 °C for 2 h. At last, a microplate reader (BioRad Laboratories, USA) was used to detect the absorbance at 450 nm wavelength [42].

### Colony formation assay

For colony formation assay, cells were transfected with the indicated constructs for 6 h and seeded into 6-well plates (1 × 10^3^ cells/well). The colonies were fixed with 4% triformol (Sigma Aldrich, USA) and stained with 0.1% crystal violet (Solarbio, Beijing, China). The colony numbers were manually counted under a stereomicroscope [43].

### Transwell assay

Cells were transfected with the indicated constructs for 72 h. Then, the cell suspension containing 1 × 10^4^ cells was seeded in the uncoated (for migration assay) or coated (for invasion assay) top chambers with Matrigel (BD Biosciences, USA). The RPMI‑1640 medium containing 20% FBS were added to the low chamber. After being incubated for 24 h, these cells were fixed with 4% triformol (Sigma Aldrich, USA) and stained with 1.5% crystal violet (Solarbio, Beijing, China) at 37°C. Migrated and invaded cells were determined and quantified with an inverted microscope (Leica, Germany) [44].

### Luciferase reporter assay

Luciferase reporter vectors, circMCTP2 wild-type (circMCTP2-WT) or mutant (circMCTP2-Mut) were cloned into pGL3-basic plasmid (Promega. USA) and co-transfected with miR-498 mimics or mimics control by using the Lipofectamine 3000 regents (Invitrogen, USA). Then, the transfected cells were harvested after transfection for 72 h. Luciferase Reporter Assay System was used to calculate the luciferase activities (Promega, USA) [45]. Each experiment was performed in triplicate.

### RNA pull-down assay

RNA pull-down assay was utilized to study the interaction between circMCTP2 and miR-498 in BC cell lines (J82 and T24). In short, for circMCTP2 pulled down miR-498, the cells were transfected with circMCTP2-WT probe or circMCTP2-Mut probe by using Lipofectamine 3000 (Invitrogen. USA) according to the supplier’s protocol. The cells were collected after transfection for 48 h and the samples were incubated with magnetic beads (Life Technologies, USA). After three washes with PBS, the mRNA level of miR-498 was calculated by using qRT-PCR assay [46].

### RNA immunoprecipitation (RIP) assay

RNA immunoprecipitation assay was utilized to confirm the relationship between miR-498 and MDM2 in BC cells. In short, cells were treated with RIP lysis buffer. The anti‑Ago2 antibody (1: 2000, Abcam, USA) or normal IgG antibody (1:2000, Abcam, USA) was conjugated to magnetic beads and incubated with the cell extract at 4°C for 24 h. Then, the magnetic beads were harvested and incubated with proteinase K. Lastly, the enrichment of miR-498 and MDM2 in immunoprecipitated RNA was assessed with qRT-PCR analysis [40].

### Xenograft tumorigenesis

The nude mice were bought from Shanghai SLAC Laboratory Animal Company (Shanghai, China). The animal research was approved by the Ethics Review Committee of The Third Affiliated Hospital of Soochow University. BC J82 cells (1 × 10^6^/mice) were transfected with sh-circMCTP2 or sh-NC suspended in PBS and injected subcutaneously into the mice. Tumor volume was measured weekly. The mice were sacrificed and the tumor tissue was weighed after 35 days [47].

## Statistical analysis

All experimental data were analyzed by Prism 7.0 software. The datum was presented as meaning ± SD. Data analyses between the two groups were evaluated using Student’s *t* test. Data analyses between multiple groups were evaluated using one-way ANOVA method. The survival curves were established using the Kaplan-Meier plot and analyzed by the log-rank test. The correlation between the expression of circMCTP2 and miR-498 in BC patient tissues was analyzed by Pearson χ^2^ tests. The significance level was set as *P* < 0.05 [48].

## Results

According to high expression of circMCTP2 in BC, we hypothesized that circMCTP2 regulates BC progression as a ceRNA and explored the molecular mechanism of it. The binding among circMCTP2, miR-498 and MDM2 was verified by luciferase reporter assay, RNA pull-down and RIP assays. Functional roles of circMCTP2/miR-498/MDM2 were investigated in J82 and T24 cells by CCK-8 assay, colony formation assay, transwell assay and western blot analysis.

### Expression levels of circMCTP2 in BC tissues and cell lines

**Figure 1.**
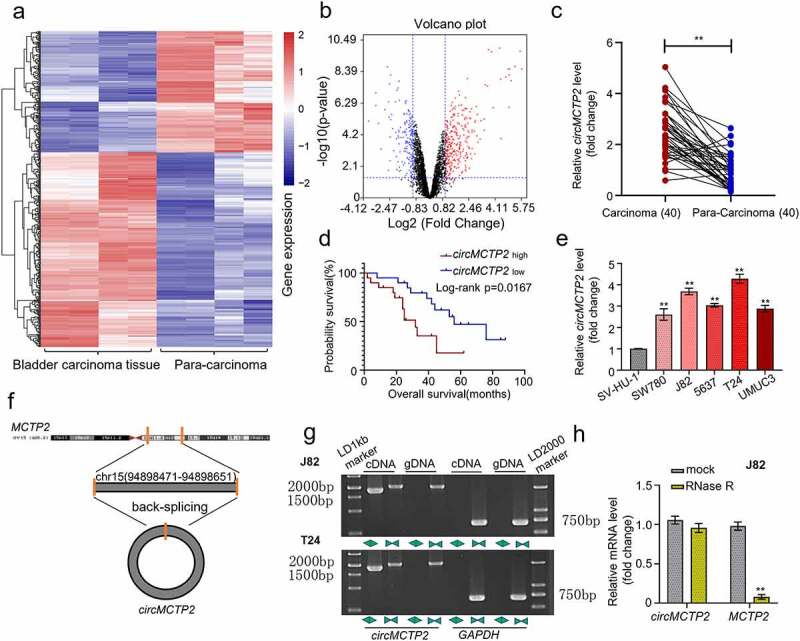
**Expression and verification of circMCTP2 in BC tissues and cell lines**. A. The heat map of differentially expressed circMCTP2 in BC a tissues and adjacent noncancerous specimens based on the GEO database. B. The volcano plot of differentially expressed circMCTP2 in BC tissues and adjacent noncancerous specimens based on the GEO database. C. The mRNA expression of circMCTP2 in 40 pairs of BC tissues and adjacent noncancerous specimens were determined by qRT‐PCR analysis. D. Kaplan-Meier survival analysis of BC patients with high or low circWHSC1 expression. E. The mRNA expression of circMCTP2 in normal urothelial cells line SV-HU-1 as well as five BC cell lines (SW780, J82, 5637, T24, and UMUC3) were quantified by qRT‐PCR analysis. F. Schematic illustration of the *MCTP2* gene forming circMCTP2 by back splicing. G. The existence of circMCTP2 was confirmed in BC cell lines (J82 and T24). H. The mRNA expression of circMCTP2 and MCTP2 in BC J82 cells with or without RNase R treatment were calculated by qRT‐PCR analysis. The experiment was performed in triplicate. ‘**’ presents *p* < 0.01.

To explore the differentially expressed circRNAs in BC, we downloaded the circRNA expression profiles from GEO database. Differential expression analysis for GEO database revealed that 435 circRNAs (168 down-regulated and 267 up-regulated) were differentially expressed in BC tissues (*n*= 4) relative to adjacent noncancerous specimens (*n* = 4) and the expression of circMCTP2 was significantly elevated in BC tumor tissues (*n* = 4) compared with adjacent noncancerous specimens (*n* = 4) (Figure 1a, b). To further confirm the elevated expression of circMCTP2 in BC, we detected the expression of circMCTP2 in 40 pairs of BC tissues and adjacent noncancerous specimens that were obtained from our hospital by qRT‐PCR assays. We found that the expression level of circMCTP2 was elevated in BC tissues relative to adjacent noncancerous specimens (Figure 1c). Base on the cutoff value, 40 patients with BC were divided into two groups (circMCTP2 high and low expression). Kaplan-Meier analysis showed that higher circMCTP2 expression was associated with lower overall survival rates of BC patients (Figure 1d). Then, we examined the expression of circMCTP2 in the normal urothelial cell line SV-HU-1 and the five BC cell lines (SW780, J82, 5637, T24, and UMUC3) by qRT‐PCR analysis. Our data demonstrated that circMCTP2 was elevated in all BC cell lines operated with the highest level in J82 and T24 cells (Figure 1e). Furthermore, our data discovered that the expression level of circWHSC1 was closely correlated with pathology stages, grades, TNM size, and lymph node metastasis as well as TNM stage (Table 1). These results indicate that circMCTP2 may participate in the development of BC.

**Table 1.** The correlation between CircMCTP2 expression and clinicopathological variables of BC patients

Clinicopathological characteristics	Total	high expression		low expression	X^2^	P value
Gender						
male	25		12	13	0.107	0.744
female	15		8	7		
Age (year)						
<65	19		11	8	0.902	0.342
≥65	21		9	12		
Pathology stage						
pTa-pT1	19		6	13	4.912	0.027
pT2-pT4	21		14	7		
Grade						
Low	20		6	14	6.400	0.011
High	20		14	6		
TNM size (cm)						
<3	17		4	13	4.912	0.027
≥3	23		16	7		
Lymph nodes metastasis						
Negative	17		5	12	5.013	0.025
Positive	23		15	8		
visceral metastasis						
Negative	19		7	12	2.506	0.113
Positive	21		13	8		
TNM stage						
I+ II	17		5	12	5.013	0.025
III+IV	23		15	8

Figure 1f indicates the schematic illustration of circMCTP2 that is originated from the MCTP2 locus in chromosome 15. Next, we were trying to amplify MCTP2 and circMCTP2 from gDNA and cDNA by using convergent and divergent primers. The result showed that circMCTP2 was only observed in the cDNA (Figure 1g). In addition, RNase R assays displayed that the linear MCTP2 was digested by RNase R, while the circMCTP2 was not (Figure 1h). These results demonstrate that circMCTP2 belong to a classical member of circular RNAs.

### CircMCTP2 deficiency impairs the cell proliferation and metastasis in BC

**Figure 2.**
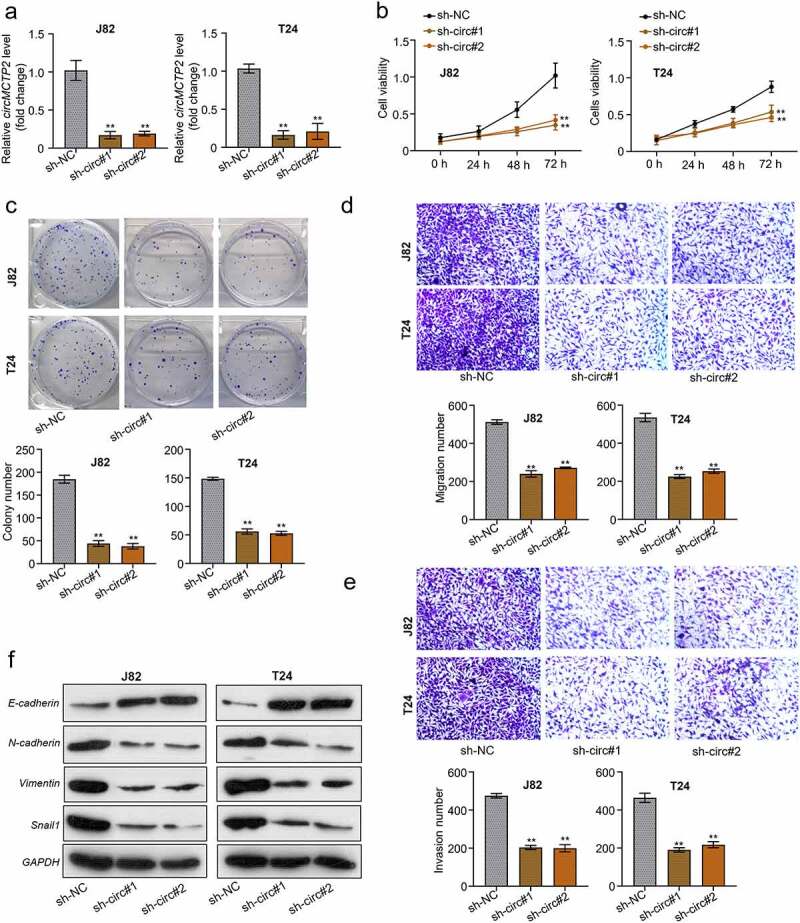
**CircMCTP2 deficiency impairs the cell proliferation and metastasis in BC**. A. The mRNA expression of circMCTP2 in BC cell lines (J82 and T24) transfected with sh-circMCTP2 (sh-circMCTP2#1 and sh-circMCTP2#2) was calculated by qRT‐PCR analysis. B. The cell viability in (a) was determined by using CCK-8 assay. C. The colony number of cell in (A) was calculated by using colony formation assay. D. The cell migration in (A) was measured by using Transwell assay (uncoated with Matrigel). E. The cell invasion in (A) was assessed by using Transwell assay (coated with Matrigel). F. The protein expression of E-cadherin, N-cadherin, Vimentin, and Snail in BC cell lines (J82 and T24) transfected with sh-circMCTP2(sh-circMCTP2#1 and sh-circMCTP2#2) was calculated by Western blot analysis. The experiment was performed in triplicate. ‘**’ presents *p* < 0.01.

To investigate the role of circMCTP2 in BC carcinogenesis and progression, we depleted the circMCTP2 in BC cell lines (J82 and T24) through transfection with sh-circMCTP2 (sh-circMCTP2#1 and sh-circMCTP2#2) (Figure 2a). Subsequently we validated the protein level of MCTP2 after knockdown by sh-circMCTP2#1 and sh-circMCTP2#2 to assess the impaction of MCTP2 itself to the alteration of phenotypes, the results showed that specific knockdown of circMCTP2 had no significant effect on MCTP2, indicating that our study was only effective for circMCTP2 (**Fig S1**). Then, we used CCK-8 assay and colony formation assay to assess the cell proliferation capability. Our results showed that circMCTP2 deficiency suppressed the J82 and T24 cell viability (Figure 2b) and decreased cell colony numbers (Figure 2c). In addition, we utilized Transwell assay to determine the metastasis capability of BC cells. Our data displayed that circMCTP2 deficiency inhibited the migration and invasion of J82 and T24 cells (Figure 2d, e). Moreover, we detected the protein expression of E-cadherin, N-cadherin, Vimentin, and Snail in BC cell lines (J82 and T24) that were transfected with sh-circMCTP2 (sh-circMCTP2#1 and sh-circMCTP2#2) by Western blot. Results elucidated that circMCTP2 deficiency elevated the protein expression of E-cadherin while diminishing the protein expression of N-cadherin, Vimentin, and Snail in BC cells (figure 2f).

### CircMCTP2 facilitates BC tumor growth in vivo

**Figure 3.**
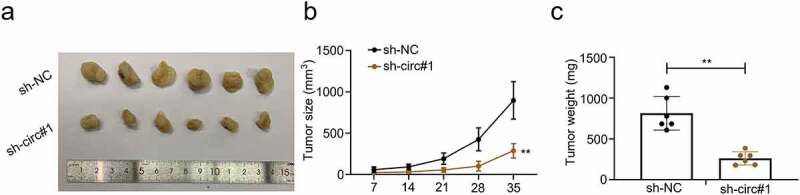
**CircMCTP2 facilitates BC tumor growth in vivo**. A. Nude mice were subcutaneously injected with the BC J82 cell transfected with sh-circMCTP2 or sh-NC. Animals were sacrificed and photographed 35 days after cell injection. B. Tumor volume was calculated at 7, 14, 21, 28, and 35 days, respectively. C. Tumor weight was calculated at the 35-day of the xenograft experiment. The experiment was performed in triplicate. ‘**’ presents *p* < 0.01.

To establish an *in vivo* model to study the function of circMCTP2 in tumor growth, nude mice were subcutaneously injected with BC J82 cells that were transfected with sh-circMCTP2 or sh-NC. Tumor volumes were evaluated 7 days after injection. The mice were sacrificed 35 days post injections. Then, subcutaneous tumor tissues were dissected, and the volume and weight of tumors were calculated (Figure 3a). Our data revealed that circMCTP2 deficiency reduced tumor growth rates (Figure 3b) and tumor weight (Figure 3c). These findings confirm that circMCTP2 acts as an oncogene in BC progression.

### CircMCTP2 serve as a sponge of miR-498

**Figure 4.**
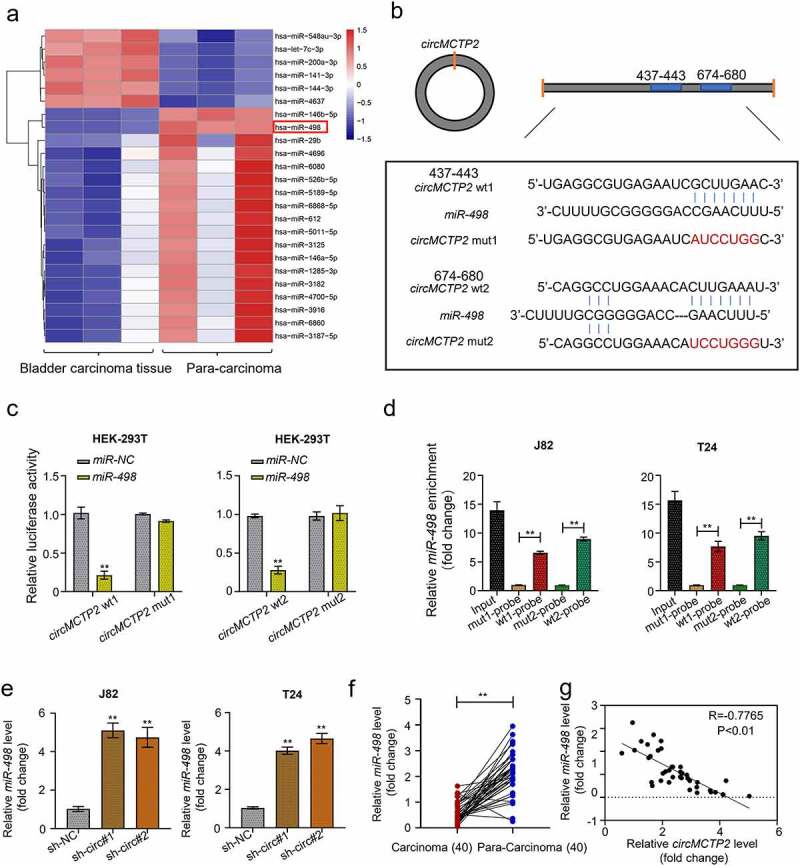
**CircMCTP2 serves as a sponge of miR-498**. A. The heat map of differentially expressed miR-498 in BC tissues and adjacent noncancerous specimens based on the GEO database. B. Potential targets of wild-type and mutant circMCTP2 on miR-498. C. The relationship between circMCTP2 and miR-498 was determined by using luciferase reporter assay. D. The relationship between circMCTP2 and miR-498 was assessed by using RNA pull-down assay. E. The mRNA expression of miR-498 in BC cell lines (J82 and T24) transfected with sh-circMCTP2 (sh-circMCTP2#1 and sh-circMCTP2#2) were calculated by qRT‐PCR analysis. F. The mRNA expression of miR-498 in 40 pairs of BC tissues and adjacent noncancerous specimens were calculated by qRT‐PCR analysis. G. The correlation between the expression of circMCTP2 and miR-498 in BC tissues was analyzed by using Pearson’s correlation coefficient. The experiment was performed in triplicate. ‘**’ presents *p* < 0.01.

CircRNAs serve as a sponge of miRNAs to regulate the expression of genes, resulting in the progression of cancers [49]. To found the targets of circMCTP2, we first mined the GEO database and found 24 miRNAs (18 down-regulated and 6 up-regulated) that were differentially expressed in BC tissues (*n* = 3) relative to adjacent noncancerous specimens (*n* = 3) (Figure 4a). Next, we predicted the putative targets of circMCTP2 from the differentially expressed miRNAs by using CircInteractome Database (https://circ-interactome.nia.nih.gov/). We concluded that that circMCTP2 could bind miR-498 (Figure 4b). To support this, we performed Luciferase reporter and RNA pull-down assays. Luciferase reporter assay elucidated that co-transfection of WT-circMCTP2, not MUT- circMCTP2, with miR-498 mimics to HEK-293 T cell reduced the luciferase reporter activity (Figure 4c). RNA pull-down assay using the biotinylated circMCTP2 probe displayed that miR-498 was detected in the circMCTP2-WT probe sample but not in the circMCTP2-Mut probe sample (Figure 4d). Furthermore, we detected the expression of miR-498 in BC cell lines (J82 and T24) transfected with sh-circMCTP2 (sh-circMCTP2#1 and sh-circMCTP2#2) by using qRT‐PCR assays. Results showed that circMCTP2 deficiency increased in expression of miR-498 in BC cells (Figure 4e). By contrast, the expression level of miR-498 was diminished in BC tissues relative to adjacent noncancerous specimens (figure 4f). In addition, our data displayed that there was a negative correlation between the expression levels of circMCTP2 and miR-498 in BC tissues (Figure 4g). Collectively, these results suggest that circMCTP2 could directly target miR-498.

### MiR-498 silencing reverses sh-circMCTP2-induced impairment of cell proliferation and metastasis in BC

**Figure 5.**
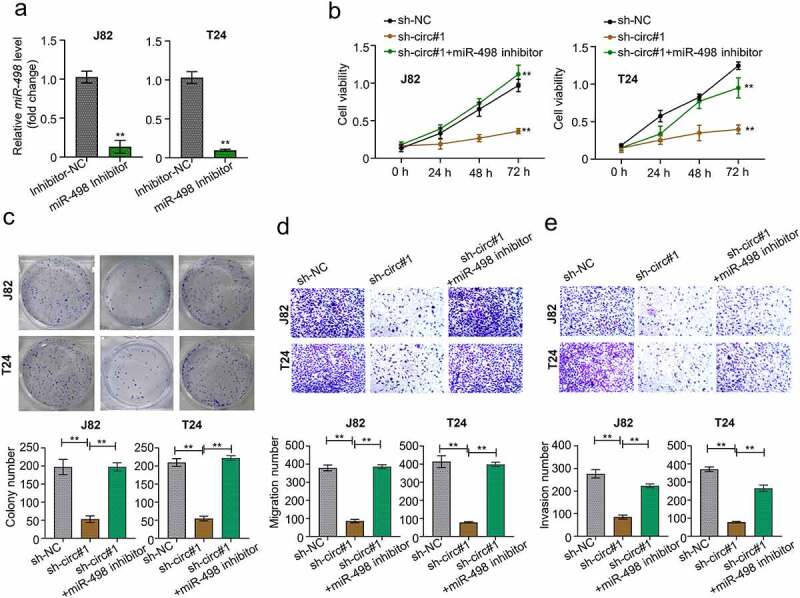
**MiR-498 silencing reverses sh-circMCTP2-induced impairment of cell proliferation and metastasis in BC**. A. The mRNA expression of miR-498 in BC cell lines (J82 and T24) transfected with miR-498 inhibitor or inhibitor control was calculated by qRT‐PCR analysis. B. The cell viability in (a) was determined by using CCK-8 assay. C. The colony number of cell in (A) was calculated by using colony formation assay. D. The cell migration in (A) was assessed by using Transwell assay (uncoated with Matrigel). E. The cell invasion in (A) was analyzed by using Transwell assay (coated with Matrigel). The experiment was performed in triplicate. ‘**’ presents *p* < 0.01.

To further prove the function of miR-498 in BC carcinogenesis and progression, we silenced the miR-498 in BC cell lines (J82 and T24) through transfection with miR-498 inhibitors (Figure 5a). Our data showed that circMCTP2 deficiency suppressed cell viability and diminished cell colony numbers, which was reversed by miR-498 silencing (Figure 5b, c). Similarly, circMCTP2 deficiency could repress the cell migration and invasion ability, which were abolished when miR-498 was silenced (Figure 5d, e). Therefore, circMCTP2 could regulate the BC progression via sponging miR-498.

### *MiR-498 directly target* MDM2 *gene*

**Figure 6.**
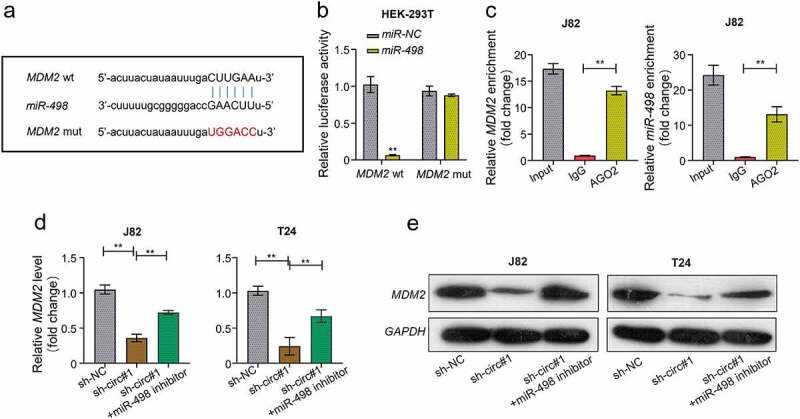
**MiR-498 could directly target the gene of *MDM2***. A. The complementary sequence of miR-498 and MDM2 was predicted by using STARBASE database. B. The relationship between miR-498 and MDM2 was assessed by using luciferase reporter assay. C. The relationship between miR-498 and MDM2 was analyzed by using RNA immunoprecipitation (RIP) assay. D. The mRNA expression of MDM2 in BC cell lines (J82 and T24) transfected with indicated constructs (sh-NC, sh-circMCTP2#1, sh-circMCTP2#1, and miR-498 inhibitor) was calculated by using qRT‐PCR analysis. E. The protein expression of MDM2 in BC cell lines (J82 and T24) transfected with indicated constructs (sh-NC, sh-circMCTP2#1, sh-circMCTP2#1, and miR-498 inhibitor) was calculated by using Western blot analysis. The experiment was performed in triplicate. ‘**’ presents *p* < 0.01.

We predicted the potential target gene of miR-498 through the online StarBase database. The prediction revealed that miR-498 and *MDM2* could form base pairings (Figure 6a). Using Luciferase reporter assay, we found that the luciferase activity was significantly decreased in HEK-293 T cells co-transfected with WT-MDM2 and miR-498 mimics, but were not when co-transfected with MUT-MDM2 and miR-498 mimics (Figure 6b). The RIP assay showed that Ago2 antibody precipitated the Ago2 protein from the cell lysates, and the expression of miR-498 and MDM2 were raised in the Ago2 pellet (Figure 6c). Moreover, our data found that both mRNA and protein levels of MDM2 expression were decreased in J82 and T24 cells transfected with sh-circMCTP2, which was reversed by miR-498 silencing (Figure 6d, e). Read in conjunction, our results support that miR-498 could directly bind to the *MDM2* gene.

### CircMCTP2 facilitates the BC cell proliferation and metastasis through regulating the miR-498/MDM2 axis

**Figure 7.**
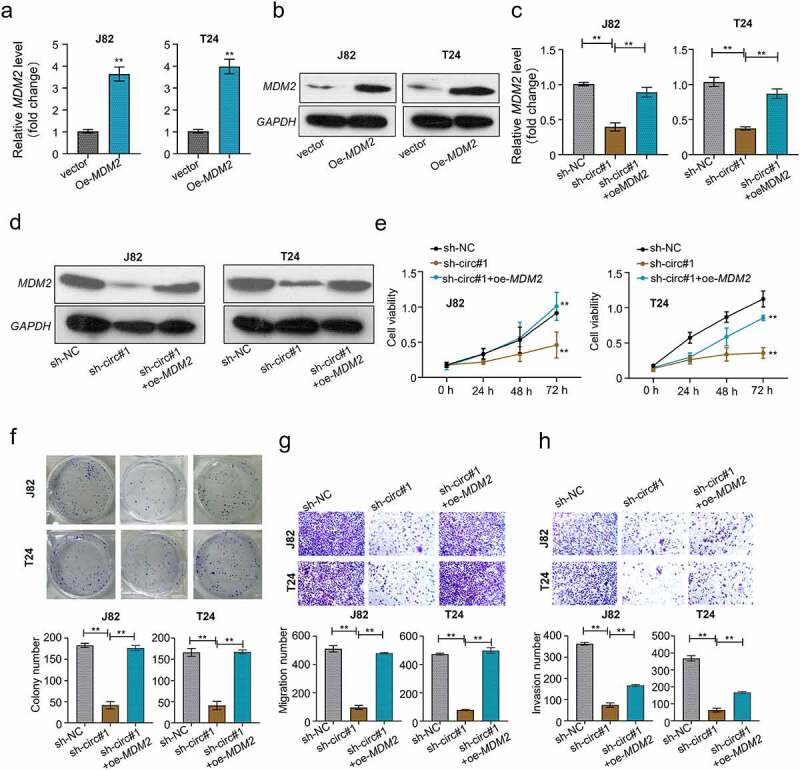
**CircMCTP2 facilitates the BC cell proliferation and metastasis through regulating the miR-498/MDM2 axis**. A. The mRNA expression of MDM2 in BC cell lines (J82 and T24) transfected with OE-vector or OE-MDM2 was calculated by using qRT‐PCR analysis. B. The protein expression of MDM2 in BC cell lines (J82 and T24) transfected with OE-vector or OE-MDM2 was calculated by using Western blot analysis. C. The mRNA expression of MDM2 in BC cell lines (J82 and T24) transfected with indicated constructs (sh-NC, sh-circMCTP2#1, sh-circMCTP2#1 and OE-MDM2) was calculated by using qRT‐PCR analysis. D. The protein expression of MDM2 in BC cell lines (J82 and T24) transfected with indicated constructs (sh-NC, sh-circMCTP2#1, sh-circMCTP2#1 and OE-MDM2) was calculated by using Western blot analysis. E. The cell viability in (d) was analyzed by using CCK-8 assay. F. The colony number of cells in (D) was calculated by using colony formation assay. G. The cell migration in (D) was measured by using Transwell assay (uncoated with Matrigel). H. The cell invasion in (D) was determined by using Transwell assay (coated with Matrigel). The experiment was performed in triplicate. ‘**’ presents *p* < 0.01.

To explore whether the circMCTP2/miR-498/MDM2 axis was involved in the BC progression. We overexpressed MDM2 in BC cell lines (J82 and T24) through transfection with MDM2 over-expression plasmid. Both mRNA and protein levels of MDM2 were elevated in J82 and T24 cells transfected with OE-MDM2 (Figure 7a, b). Conversely, both mRNA and protein levels of MDM2 were diminished in J82 and T24 cell transfected with sh-circMCTP2, which was reversed by the transfection of OE-MDM2 (Figure 7c, d). Moreover, circMCTP2 deficiency inhibited the cell proliferation, colony formation, migration, and invasion ability, an effect that was abrogated by co-transfection with OE-MDM2 (Figure 7e-h). These results suggest that circMCTP2 facilitates the proliferation and metastasis of BC cells through regulating the miR-498/MDM2 axis.

## Discussion

BC is a frequent malignant tumor with approximately 75% of new cases being non-muscle-invasive and about 25% being muscle invasive BC [50]. According to the global cancer statistics, 5-year overall survival rate of patients with non-muscle-invasive is approximately 90%, whereas the rate of the ones with muscle invasive bladder cancer is only 60% [51,52]. Although many advances in therapeutic strategy have been made in the treatment of BC, the 5- year survival rate of BC patients and prognosis remain insignificant [5]. Thus, exploring the molecular mechanisms underlying the progression and tumorigenesis of BC is useful to formulate a better therapeutic strategy and improve the prognosis of BC patients.

CircMCTP2 is originated from the MCTP2 locus that is situated on chromosome 15. CircMCTP2 could repress gastric cancer cisplatin resistance via regulating the miR-99a-5p/MTMR3 signaling, revealing that circMCTP2 might be important for malignant tumor progression [21]. In this study, we found that circMCTP2 expression was significantly elevated in BC tumor tissues compared with adjacent noncancerous specimens (Figure 1a). Further analyses confirmed that the expression of circMCTP2 was elevated in BC tissues and cell lines (Figure 1c, e). Moreover, high expression of circMCTP2 predicted a poor prognosis of the patients with BC (Figure 1d). Apart from that, we performed qRT‐PCR and RNase R assay to verify the stable structure of circMCTP2. Our data showed that circMCTP2 was only found in the cDNA and the linear MCTP2 was digested with RNase R, while the circMCTP2 was not (Figure 1h). In addition, our results found that circMCTP2 deficiency impaired the cell proliferation and metastasis abilities *in vitro* and suppressed the BC tumor growth *in vivo* (Fig 2a-f, Fig 3a-c). Collectively, our studies show the importance of circMCTP2 in the progression of malignant cancers.

Accumulating evidence indicates that circRNAs regulates the malignant tumor progression through sponging miRNAs [53–55]. Built on the differential expression analysis of GEO database, we found that miR-498 might be the target of circMCTP2 (Figure 4a). By Luciferase reporter and RNA pull-down assays, we confirm that circMCTP2 directly target the miR-498 (Figure 4c-d). MiR-498 has been involved in the progression of diverse tumors, such as hepatocellular cancer, lung cancer, and colon cancer [24–26]. However, the function of miR-498 in the development of BC remains unknown. In our study, we found that circMCTP2 deficiency elevated the expression of miR-498 in BC cell and the expression of miR-498 was diminished in BC tissues (Figure 4f). Our data also noted that there was a negative correlation between the expression levels of circMCTP2 and miR-498 in BC tissues (Figure 4g). In addition, our results showed that circMCTP2 deficiency impaired the cell proliferation and metastasis ability in BC, which was stopped by miR-498 silencing, implying that miR-498 could eliminate the oncogene effect of circMCTP2 in BC.

It has been reported that miRNAs bind their target genes, thereby suppressing the gene expression [56]. In our study, we confirmed that miR-498 directly targeted the gene of *MDM2* by Luciferase reporter and RIP assays (Figure 6a-c). Moreover, circMCTP2 deficiency could diminish both mRNA and protein expression of miR-498 in BC cells (Figure 6d, e). MDM2 is a p53-binding protein that induces the degradation of p53 protein [27,28], which is in line with a previous study that reveals that MDM2 is an oncogene in the progression of malignant tumor [32]. Moreover, it has be shown that lncRNA-LOC572558 repress the progression of BC through modulating the AKT-MDM2-p53 signaling pathway [57]. OCT3/4 enhances the immune response of BC tumors through regulating the NRF2/MDM2 signaling pathway [34]. Other than that, MDM2 is associated with the invasive growth of BC [58]. These studies clearly support that MDM2 is a versatile protein functioning in multiple signaling pathway to influence the progression of tumors. Here, our data revealed that circMCTP2 deficiency impairs the cell proliferation and metastasis abilities *in vitro*, an effect that was removed by the co-transfection with OE-MDM2 plasmid, suggesting that circMCTP2 facilitates the BC cell proliferation and metastasis through regulating the miR-498/MDM2 axis.

## Conclusion

In sum, we first elucidate that circMCTP2 expression was high in BC tissues and cell lines. Next, we discovered that circMCTP2 could govern the expression of MDM2 by sponging miR-498 to promote the development of BC. Our findings not only gain new insight into the progression of BC but also provide a potential strategy for early diagnosis and therapeutics.

## Supplementary Material

Supplemental Material

## Data Availability

The datasets used and/or analysed during the current study are available from the corresponding author on reasonable request.
