# Supplementary figures and images for: CircMCTP2 (has-circ-0000658) facilitates the proliferation and metastasis of bladder carcinoma through modulating the miR-498/murine double minute-2 axis
Source: Bioengineered. 2022 Apr 27;13(4):10734–48. doi: 10.1080/21655979.2022.2054161 (PMC9208511; doi:10.1080/21655979.2022.2054161)

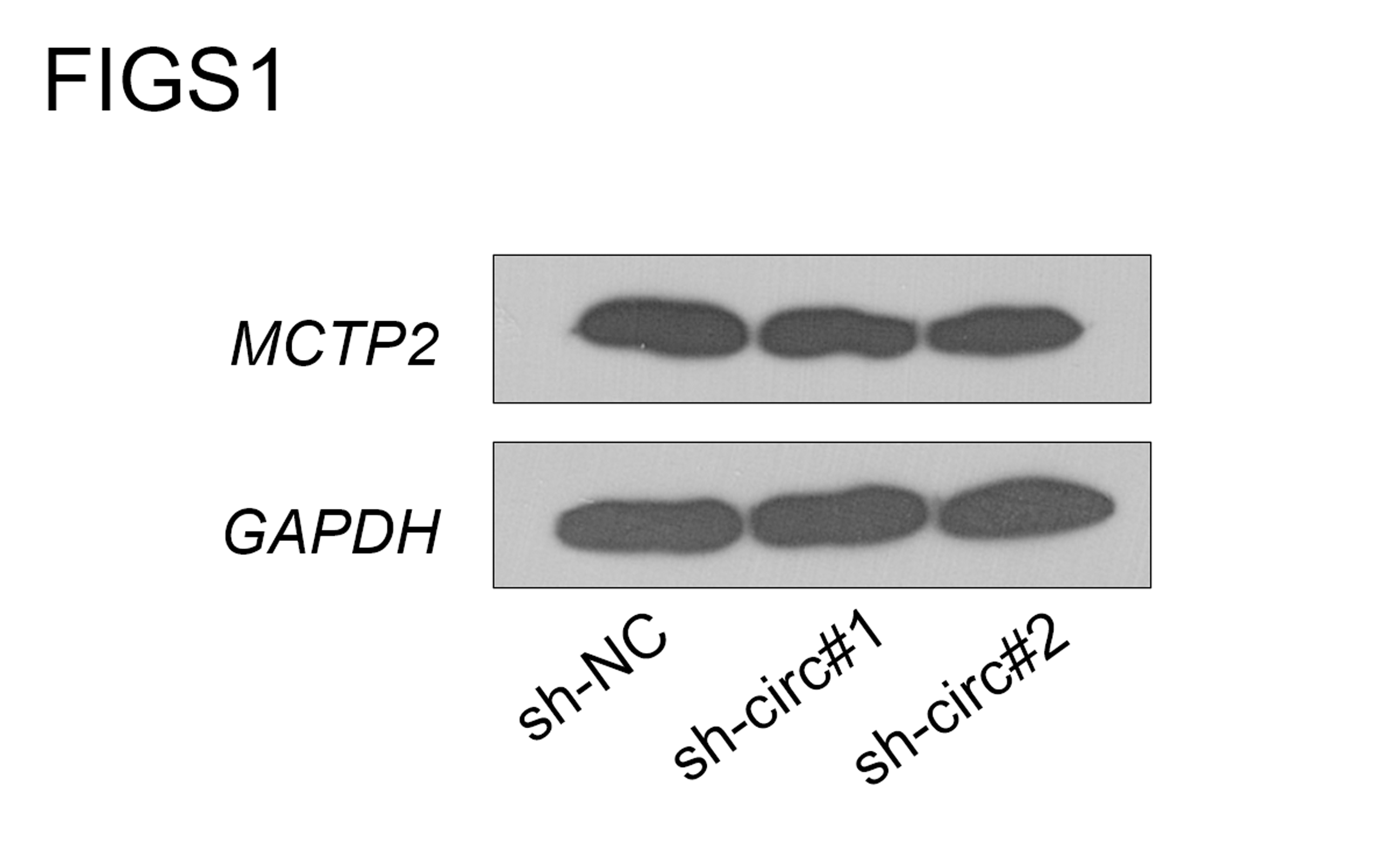

Supplement: Supplemental Material [file KBIE_A_2054161_SM0167.tif]
